# Isolated anterior pituitary dysfunction in adulthood

**DOI:** 10.3389/fendo.2023.1100007

**Published:** 2023-03-08

**Authors:** Nunzia Prencipe, Lorenzo Marinelli, Emanuele Varaldo, Daniela Cuboni, Alessandro Maria Berton, Fabio Bioletto, Chiara Bona, Valentina Gasco, Silvia Grottoli

**Affiliations:** Department of Medical Science, Division of Endocrinology, Diabetes and Metabolism, University of Turin, Turin, Italy

**Keywords:** hypopituitarism, traumatic brain injury, radiotherapy, hypocortisolism, hypothyroidism, hypogonadism, hypoprolactinemia, growth hormone deficiency

## Abstract

Hypopituitarism is defined as a complete or partial deficiency in one or more pituitary hormones. Anterior hypopituitarism includes secondary adrenal insufficiency, central hypothyroidism, hypogonadotropic hypogonadism, growth hormone deficiency and prolactin deficiency. Patients with hypopituitarism suffer from an increased disability and sick days, resulting in lower health status, higher cost of care and an increased mortality. In particular during adulthood, isolated pituitary deficits are not an uncommon finding; their clinical picture is represented by vague symptoms and unclear signs, which can be difficult to properly diagnose. This often becomes a challenge for the physician. Aim of this narrative review is to analyse, for each anterior pituitary deficit, the main related etiologies, the characteristic signs and symptoms, how to properly diagnose them (suggesting an easy and reproducible step-based approach), and eventually the treatment. In adulthood, the vast majority of isolated pituitary deficits are due to pituitary tumours, head trauma, pituitary surgery and brain radiotherapy. Immune-related dysfunctions represent a growing cause of isolated pituitary deficiencies, above all secondary to use of oncological drugs such as immune checkpoint inhibitors. The diagnosis of isolated pituitary deficiencies should be based on baseline hormonal assessments and/or dynamic tests. Establishing a proper diagnosis can be quite challenging: in fact, even if the diagnostic methods are becoming increasingly refined, a considerable proportion of isolated pituitary deficits still remains without a certain cause. While isolated ACTH and TSH deficiencies always require a prompt replacement treatment, gonadal replacement therapy requires a benefit-risk evaluation based on the presence of comorbidities, age and gender of the patient; finally, the need of growth hormone replacement therapies is still a matter of debate. On the other side, prolactin replacement therapy is still not available. In conclusion, our purpose is to offer a broad evaluation from causes to therapies of isolated anterior pituitary deficits in adulthood. This review will also include the evaluation of uncommon symptoms and main etiologies, the elements of suspicion of a genetic cause and protocols for diagnosis, follow-up and treatment.

## 1 Background

Hypopituitarism is defined as a complete or partial deficiency in one or more pituitary hormones. It can be a result of diseases that either reduce or abolish the pituitary function or which can interfere with pituitary stalk integrity or the hypothalamic secretion of pituitary-releasing hormones. The prevalence of hypopituitarism is approximately 45 cases per 100.000 and the incidence of about 4 cases per 100.000 per year (1). Anterior hypopituitarism can include central adrenal insufficiency (CAI), central hypothyroidism (CH), hypogonadotropic hypogonadism (HH), growth hormone deficiency (GHD) and seldom hypoprolactinemia. Patients with hypopituitarism suffer from an increased disability and sick days, resulting in lower health status, higher cost of care and an increased mortality (2).

Pituitary deficits usually present as combined; in adulthood, however, it is not uncommon to develop isolated pituitary deficits as well. This often becomes a diagnostic challenge for the physicians due to several reasons: the clinical picture may be vague and baseline and dynamic hormonal assessments may not be fully reliable. For these reasons, a considerable proportion of isolated pituitary deficits still remains without a defined cause and thus precise data on their prevalence and incidence are lacking.

The aim of this narrative review is to analyse the main etiopathogenetic processes that lead to isolated anterior pituitary deficits, focusing on their signs, symptoms, hormonal evaluations, and treatments. It is important to remark that there is a lack of guidelines for the specific management of isolated anterior pituitary deficits; as such, part of the evidence related to diagnosis and management of these conditions derives from current knowledge regarding hypopituitarism.

## 2 Common and shared causes of isolated pituitary deficiencies

Before discussing the specific causes linked to each anterior pituitary deficit, it is pivotal to describe the most common causes that can induce isolated deficits in adulthood (**Table 1**). Among them, pituitary region masses (tumors and infiltrative diseases), treatments as neurosurgery and radiotherapy, autoimmune diseases and traumatic brain injuries (TBI) are surely to be considered. Genetic causes generally determine isolated deficits with an onset early in childhood, but HH and CH can be linked to altered genes that can manifest even later in life (3, 4).

**Table 1:** Main causes of isolated pituitary deficits.

CAUSES OF ISOLATED PITUITARY DEFICIENCIES
ACQUIRED CAUSES
***Pituitary tumors:*** • Hormone-secreting adenoma • Non-functioning adenoma
***Non pituitary tumors:*** • Rathke’s cleft cyst, Craniopharyngioma, Meningioma, Chordoma, Astrocytoma, Ependymoma, Germinoma, Lymphoma, others • Metastases
***Oncological treatments:*** • Neurosurgery • Irradiation • Chemotherapy
***Infiltrative disorders:*** • Sarcoidosis, Wegener granulomatosis, Haemochromatosis, Langerhans cell Histiocytosis, Erdheim–Chester disease
***Autoimmune diseases:*** • Primary hypophysitis (lymphocytic, granulomatous, xanthomatous, necrotizing) • Drug induced hypophysitis (Immune checkpoint inhibitors: PD1, PDL1, CTLA4 inhibitors)
***Traumatic brain injuries and vascular damage:*** • Traumatic brain injury • Sub-arachnoid haemorrhage • Stroke • Pituitary apoplexy • Sheehan syndrome
***Drugs***
***Idiopathic***
GENETIC CAUSES

### 2.1 Pituitary tumors

Pituitary dysfunction, as a consequence of intrasellar tumor masses originating from the pituitary or Rathke’s pouch, is a well-known problem. Sellar masses are often associated with combined pituitary deficits, and these are induced by the tumor itself or by further damage secondary to surgery, radiotherapy, or medical therapies. However, at diagnosis no hormonal deficits can be reported and they may develop only during the follow-up, generally as a consequence of the tumor growth (5).

### 2.2 Irradiation

**Figure 1:**
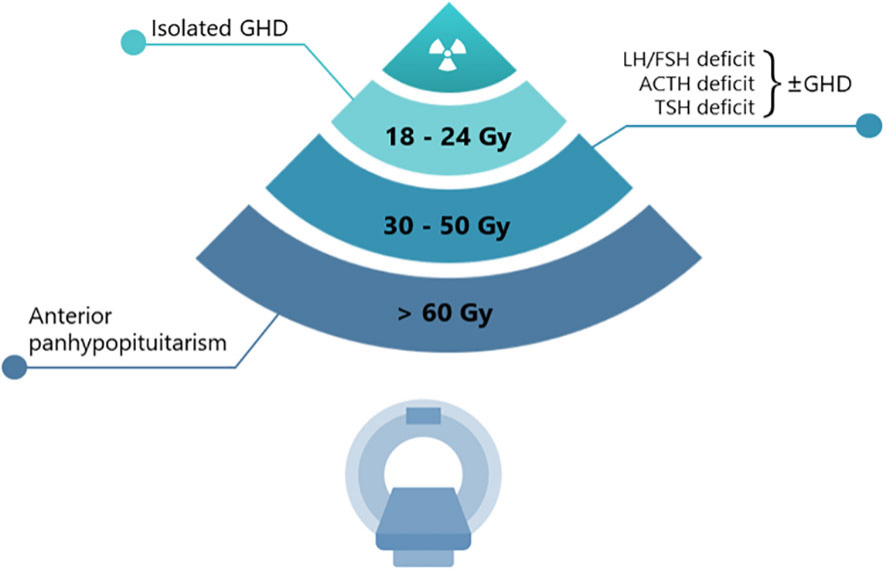
Radiation progressive damage of pituitary function.

The potential damage induced by radiotherapy is usually confined to the anterior pituitary gland. The sensitivity to radiation is quite different for each hypothalamic-pituitary axis (**Figure 1**). The GH (growth hormone)-secreting cells are the most sensitive, followed by the FSH (Follicle-Stimulating Hormone)- and LH (Luteinizing Hormone)-secreting cells, while those secreting ACTH (Adrenocorticotropic Hormone) and TSH (Thyroid Stimulating Hormone) are the most resistant (6, 7). In agreement with these observations, a single dose of 3 Gray (Gy) has been reported to impair the *in vitro* secretion of GH (and prolactin) by pituitary cells, whereas TSH-secreting cells have proven to be resistant to doses higher than 10 Gy (8). Although lower than conventional radiotherapy, the risk of onset of hypopituitarism needs to be considered also in patients undergoing sellar/parasellar post-surgery gamma knife radiosurgery (GKRS), with an approximate incidence rate of hypopituitarism of 18–32% both for non-secreting and secreting adenomas (9, 10). The delayed onset of post-GKRS hypopituitarism has been reported in a few studies, approximately occurring 22–25 months after treatment and with an increased rate over time (11).

### 2.3 Other than pituitary tumors

Pituitary dysfunction can also be the result of brain damage due to extra-pituitary tumors (and their surgical treatment). For example, an emerging clinical entity is primary lymphoma, localized in the sellar region (12). However, after a long-term disease remission, patients who suffered from lymphoma can experience gradual reverse of hypopituitarism up to a residual isolated pituitary deficit (13). Moreover, chemotherapy (14) or irradiation might cause additional pituitary insufficiencies. In their study, Schneider et al. (15) recorded isolated pituitary hormone deficits in 16.2% of patients diagnosed and treated for an extra-sellar tumor. Endocrine deficiencies may become evident from 3 months to more than 10 years after irradiation (7).

### 2.4 Infiltrative diseases

Rare causes of hypopituitarism include infiltrative disorders such as neurosarcoidosis, Wegener granulomatosis, hemochromatosis, Langerhans cell histiocytosis and Erdheim–Chester disease. Diabetes insipidus usually represents the onset of these diseases; however, in all unknown cases of anterior hypopituitarism they need to be ruled out (16).

### 2.5 Autoimmune disease and drug induced autoimmune disorders

Based on histological features, primary hypophysitis is classified as lymphocytic, granulomatous, xanthomatous or necrotizing inflammation (17, 18). Lymphocytic hypophysitis is the most frequent (19) and it is caused by immune-mediated diffuse infiltration of lymphocytes and plasma cells. IgG4-related hypophysitis is a recently discovered subtype of autoimmune disorder associated with multiorgan IgG4-related systemic disease (IgG4-RD). Pituitary involvement of IgG4-RD had initially been considered rare, but more recent studies (20) suggested that its prevalence is underestimated. In these conditions, isolated anterior pituitary hormone deficiencies and/or central diabetes insipidus could be observed. Pituitary inflammation may also be secondary to several kinds of drugs. Although various forms of drug-induced hypophysitis have been reported, with the inclusion of immunotherapy for many types of cancer, immune checkpoint inhibitor (ICPi)-induced hypophysitis has recently emerged as a not uncommon immune-related adverse event. Interestingly, relatively specific hormone deficiency has been detected in these cases, as described in the following paragraphs (21).

### 2.6 Traumatic brain injuries, vascular damage and infective diseases

Pituitary hormone deficiency is frequent among TBI survivors; in fact, 40–50% of patients studied suffered from some degree of pituitary dysfunction (22–24). In particular Steven et al. (23) reported a prevalence of 51.4% of isolated deficits in TBI patients, while Kelly et al. (24) reported that somatotroph and gonadotroph deficiencies were the most common and that diffuse brain swelling, hypotensive or hypoxic insults, and a relatively low Glasgow Coma Scale (GCS) score were associated with pituitary insufficiency. Because the majority of TBI survivors are young adults with near-normal life expectancy, the implications of undiagnosed post-traumatic pituitary failure can be serious and may contribute to the significant morbidity associated to it.

Among the causes of vascular damage which can potentially lead to pituitary deficits are to be counted subarachnoid haemorrhage (SAH), aneurysms, pituitary apoplexy, Sheehan syndrome and rarer infective causes such as tuberculosis, neurosyphilis, and snake bite (25, 26). In particular, it must be considered that pituitary masses, in particular non-functioning adenomas, are susceptible to haemorrhages and infarction (pituitary apoplexy). This can occur if an imbalance between oxygen or metabolic demand and available supplies happens (27). Surgeries (in particular cardiac and orthopaedic), arterial pressure fluctuations, micro-embolism, cranial trauma, dynamic pituitary testing [in particular insulin tolerance test (ITT), TRH (thyrotropin releasing hormone) and GHRH (Growth Hormone Releasing Hormone) tests], anticoagulant and to a lesser extent dopamine-agonist therapy are the main factors which can predispose to pituitary apoplexy. Hypopituitarism can be an early result of these phenomena in 15 to 85% of patients (28). The Sheehan syndrome represents a particular subtype of pituitary apoplexy; this can occur in pregnant women who suffer from an extensive uterine bleeding during the peri- or post-partum periods. This results in a pituitary infarction that leads to progressive pituitary function deficit up to a definitive hypopituitarism and a radiological “empty sella” (29, 30). It is notable to mention that an increased risk of cerebral and subsequent pituitary haemorrhage can be related to congenital conditions of increased spontaneous bleeding. This being said, hemophilic patients are considered at potential higher risk of pituitary dysfunction (31, 32) due to a significant prevalence of intracranial haemorrhage, particularly in children (33).

### 2.7 Drugs

Several drugs can lead to transient or permanent hypopituitarism. In some cases, the pituitary deficits can be isolated due to the involvement of a single axis. In fact, physio-pathological mechanisms are different for each drug and for each involved pituitary hormone. A more detailed discussion is present in the dedicated paragraph of the isolated deficit.

### 2.8 Genetic causes

In embryonic life, pituitary gland development is a result of a specific sequence of genes that express certain transcription factors (34). Particular genetic mutations can lead to a certain degree of hypopituitarism. In particular, each of the isolated pituitary deficits analysed in this review can find in genetics a possible etiology. Considered to be congenital, the clinical picture usually manifests at birth or in early childhood. The clinical phenotype can include also somatic alterations (35). However, we are going to further discuss potentially involved genes in each of the following dedicated sections.

## 3 Isolated central adrenal insufficiency

### 3.1 Definition and epidemiology

Isolated central adrenal insufficiency (ICAI) is a rare disorder, first reported by Steinberg in 1954, characterized by low or absent cortisol production and normal secretion of other pituitary hormones (36). The prevalence of CAI, most commonly due to exogenous glucocorticoid administration, is much higher than that of primary adrenal insufficiency (PAI), with an estimate of 150–280 cases per million (37). Due to its rarity, however, epidemiology and etiology of ICAI still remain uncertain.

ICAI is classified into congenital and acquired forms. In neonatal or childhood ICAI a genetic origin may be suspected, while in adults the main cause is the prolonged use of synthetic glucocorticoids (sGC). In fact, sGC are widely used for their anti-inflammatory and immunosuppressive actions and an undesirable effect of sGC treatment is, indeed, suppression of the hypothalamic-pituitary-adrenal (HPA) axis, which can lead to ICAI. A systematic review and meta-analysis found that this is a frequent scenario which could happen in 48.7% of cases, with the highest risk in hematologic patients (60%), in kidney transplant (56.2%), inflammatory bowel disease (52.2%), and rheumatologic disorders (39.4%). Risk factors for sGC-induced ICAI include the duration of glucocorticoid therapy, the dose, potency, route of administration and individual susceptibility (38). Moreover, an emerging cause of ICAI is related to an autoimmune response, due to drugs administration, such as CTLA4 (Cytotoxic T-Lymphocyte Antigen 4) and PD1/PDL1 (Programmed Cell Death Protein 1/Programmed Death Ligand 1) inhibitors. Hypophysitis appears more often in men older than 60 years of age and it is 2–5 times more frequent than in women. The reported incidence is 4%–20% with ipilimumab, 0.6% with nivolumab, 0.7% with pembrolizumab and slightly more commonly (8-10%) with combination ipilimumab and nivolumab (21, 39). Of note, these drugs could also be responsible for PAI with an incidence of less than 1% with monotherapy and 4%–8% with combined immunotherapy (40). In this clinical setting ACTH measurement can be useful for a proper differential diagnosis.

CAI is common in ICPi-related hypophysitis and, while CH and HH may be transient and spontaneously recover, ICPi-related CAI appears to be permanent in most cases resulting as an isolated deficiency.

### 3.2 When to suspect an isolated central adrenal insufficiency

CAI has several clinical presentations and, if unrecognized, a potentially fatal course. Usually, clinical manifestations of ICAI are similar to those found in pan-hypopituitarism and PAI although, generally less severe (41).

Patients with ICAI usually feel relatively well during unstressed periods until certain events trigger an acute adrenal crisis. This serious condition is characterized by extreme fatigue, severe hypotension and hypoglycemia, fever, acute abdominal pain, nausea, vomiting and diarrhoea and, if not promptly recognized, it may be irreversible and lethal. Except for adrenal crisis, patients with ICAI usually present with non-specific symptoms, such as asthenia, anorexia, unintentional weight loss and tendency to hypoglycemia. Unlike PAI, both hyperpigmentation and symptoms and signs of mineralocorticoid deficiency (e.g., salt-craving, postural hypotension) are absent. Hyponatremia may occasionally occur as the result of the increased antidiuretic hormone secretion, due to higher levels of corticotropin releasing factor (CRF). Under normal circumstances, cortisol suppresses both production of CRF and vasopressin (AVP) in the hypothalamus. In ICAI, persistently low concentrations of cortisol fail to suppress AVP and hyponatremia results from impaired free-water excretion, as it happens in the syndrome of inappropriate antidiuresis (SIAD) (42).

Another interesting clinical scenario of isolated ACTH deficiency is the so-called “critical illness-related corticosteroid insufficiency (CIRCI)” which is a condition that may develop in prolonged critically ill patients. When patients remain dependent on vital organ support for weeks, they are at risk of acquiring ICAI. This situation is determined by the increase in the systemic availability of glucocorticoids, mainly due to the reduction of circulating cortisol-binding proteins; the resulting transient elevation in serum free cortisol values exerts a negative feedback mechanism at the hypothalamus and pituitary level on the HPA axis. Additionally, elevated levels of other glucocorticoid receptor ligands (such as bile acids) and drugs (such as opioids) can further suppress ACTH secretion. The adrenal cortex, deprived for weeks of the trophic stimulus due to the ACTH signalling, can become structurally and functionally impaired, resulting in insufficient cortisol secretion. This HPA axis suppression can be maladaptive and contributes to the persistent need for vasopressors and to the development of encephalopathy, thus reducing the chances of recovery (43).

Besides typical symptoms of CAI, in Literature several cases of atypical ICAI presentation are reported as well, like flexion contractures of the legs (44), severe muscle atrophy (45) and rhabdomyolysis (46). Other unusual presentations are pericardial effusion (47), recurrent syncope (48), cholestatic jaundice (49) and petrified ear auricles (50). These clinical manifestations, though not typical of ICAI, seem to be closely related to the hypoadrenal condition because they disappear upon the start of steroid replacement therapy. Primary infertility (51), Crohn’s disease (52), myasthenia gravis (53), polycystic kidney disease (54), spinocerebellar ataxia type 3 (55), benign endocranic hypertension (56), Down syndrome (57), cognitive impairment and mental health disorders (58, 59), chronic opiate use (60) and haemodialysis (61) have also been reported in conjunction with ICAI, as well as paraneoplastic syndrome (55).

Lastly, ICAI is not infrequent in chronic alcoholism (62) and, as in patients with the above-mentioned disorders, it should be considered if more typical hypoadrenal symptoms are present. Likewise, ICAI should also be kept in mind in patients with other autoimmune diseases: ICAI due to lymphocytic hypophysitis has often been described associated with autoimmune hypothyroidism (63, 64) and, occasionally, with Graves’ disease (65), type 1 diabetes mellitus (66) and polyglandular autoimmune failure (67). Recently Morita et al. described a case of ICAI following immunization with the BNT162b2 SARS-CoV-2 vaccine (68).

### 3.3 Diagnosis

Guidelines addressing the specific management of ICAI, as of today, are lacking. Despite this, the diagnostic and therapeutic process can be ascribed to the non-isolated form. The diagnosis of CAI is mainly based on measurement of morning serum cortisol. In fact, cortisol level <3 µg/dL is indicative of adrenal insufficiency (AI) and a cortisol level >15 µg/dL likely excludes an AI diagnosis (69). In addition, guidelines suggest performing a corticotropin stimulation test when morning cortisol values are between 3 and 15 µg/dL to diagnose AI. Peak cortisol levels <18.1 µg/dL at 30 or 60 minutes indicates AI. Both 250 µg and 1 µg stimulation tests could be used (70). In fact, since after the injection of 250 µg supra-physiological concentration of corticotrophin is achieved, a stimulation with 1 µg dose was proposed (71). However, In CAI before adrenal atrophy has occurred, the sensitivity of both tests may be low (72). Some Centres propose metyrapone test to evaluate the response to drug-blocking 11 beta hydroxylase. In fact, the administration of metyrapone should cause a reduction in blood cortisol levels, further incrementing ACTH, and finally an increase of adrenal steroids synthesis of 11- deoxycortisol, measured during the test (73). Despite the interesting pathophysiological basis, the analysis of 11-deoxycortisol levels is not available in most laboratories. The Glucagon (GST) test is another alternative. Although it has been used more frequently in the evaluation of GH axis, GST offers an opportunity to assess both the HPA and GH axes, making it an interesting possibility (74). However, the proper establishment of cut-off levels of cortisol is needed to properly interpret the results. The insulin tolerance test (ITT) is another option for CAI diagnosis, although it is contraindicated in some clinical conditions, it is unpleasant for patients, and requires close medical supervision (75). To avoid performing ITT, Gasco et al. in their study aimed to detect the morning serum cortisol cut-off with a specificity or a sensitivity above 95% that could identify those patients who should not be tested with ITT, finding that the cut-off of morning serum cortisol concentration that best predicted a normal response to ITT was >16.11 µg/dL (76). Moreover, a multiparametric score has been recently proposed for the prediction of CAI when morning cortisol is in the grey zone; this score might be helpful for a finer tailoring of the diagnostic process, as it might avoid the execution of a stimulation test in approximately one-fourth of the patients in which morning cortisol values are ‘per se’ non-diagnostic (77). Although the medical history and some symptoms could help the clinician in discerning ICAI from PAI, in dubious cases, ACTH measurement is also recommended (78).

In patients without any pituitary deficit before surgery, it is necessary to assess the HPA axis as early as possible in the post-operative phase, while the evaluation of the other axes (thyroid, gonadal and growth hormone function) can be carried out later. The indications for the diagnosis of ICAI in the early post-pituitary surgery period are slightly different. In this setting, there are not clear recommendations (69); in spite of that it would be cautious to assess adrenal function on the second day after pituitary surgery, using cut-off values that international guidelines suggested for non-stressed conditions (69, 78). In fact, second-day cortisol levels ≤3.2 μg/dL and >14 μg/dL would be diagnostic of ICAI and normal HPA function, respectively (79).

**Figure 2:**
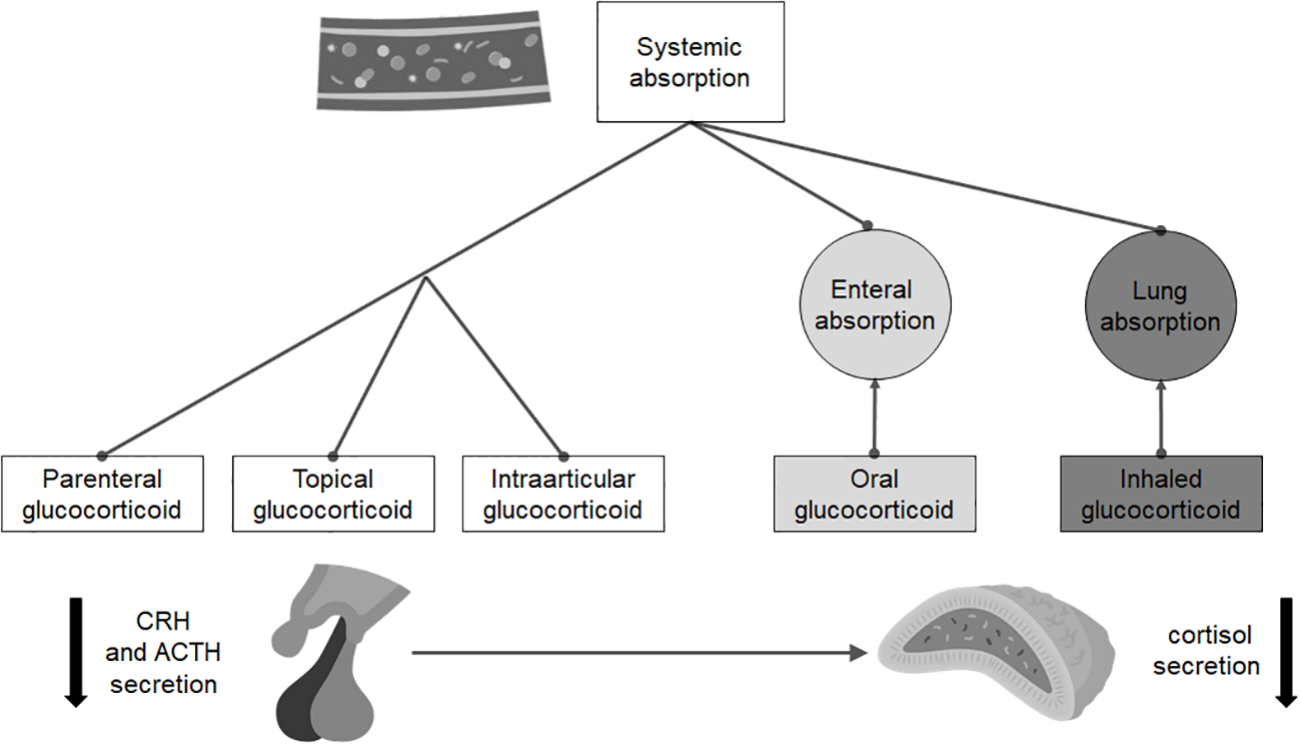
Routes of administration and absorption of the main steroid drugs that can induce isolated adrenal insufficiency.

For the diagnosis of sGC-induced ICAI (**Figure 2**), the indications are, again, different. Since the risk of developing it mainly depends on the molecule and the duration of therapy, the recovery of ICAI should be assessed only in patients taking a prednisolone dose equivalent or inferior to the replacement one (i.e., doses of prednisone no higher than 5 mg a day). Several approaches can be used, but all evaluations require at least a 24-hour wash-out of any sGC therapy in place: the most common strategies include measurement of morning cortisol concentration, synthetic ACTH stimulation tests, metyrapone test and ITT. Recently, Prete and Bancos proposed a risk guided algorithm for the recovery evaluation of HPA axes (80).

In general, once the diagnosis of ICAI has been established, a magnetic resonance imaging (MRI) of the hypothalamic-pituitary region is mandatory. In patients with autoimmune endocrine disorders pituitary antibodies should be assessed, especially in case of consistent radiological findings. Genetic testing has so far proven to be of little use in adult ICAI.

### 3.4 Therapy and follow-up

Before starting a proper replacement therapy, clinicians should carefully assess the presence of ICAI (excluding combined pituitary deficiencies) in order to deliver the most adequate therapy (69). The latest hypopituitarism guidelines recommend using hydrocortisone, usually 15–20 mg total daily dose in single or divided doses as the proper replacement therapy.

It is possible that some patients are suffering from ICAI and epilepsy (or taking preventive anti-epileptic therapy) at the same time (e.g., TBI/SAH); in these patients it is necessary to take into account the possible need for higher doses of hydrocortisone replacement treatment (69).

Clinicians should appropriately warn patients about stress-dose and emergency sGC administration and suggest obtaining an AI emergency card and an emergency kit containing injectable high-dose sGC. Lastly it is advisable not to use fludrocortisone in patients with ICAI (69). As for PAI, guidelines recommend that clinicians treat patients with suspected adrenal crisis due to ICAI with an immediate parenteral injection of 50 –100 mg hydrocortisone.

Once ICAI is diagnosed and replacement treatment is started, the patient’s follow-up includes periodic evaluation of AI symptoms and assessment of serum sodium, potassium and glucose. In the same way, follow-up of patients at risk of developing ICAI over time needs to be more standardized since, in this setting, there are different and not univocal recommendations.

For example, in all patients that previously underwent to brain/pituitary irradiations (cranial RT at doses > 30 Gy) or sellar or suprasellar region surgeries, who are at risk for developing ICAI, an annual evaluation of morning cortisol blood concentration should be performed (81).

In patients with a history of TBI, signs and symptoms possibly related to life-threatening adrenal deficiency must be immediately investigated whenever they become apparent. Alternatively, some authors suggest an endocrine evaluation performed routinely at a period between 6 and 12 months after TBI (82).

During treatment with some CTLA-4/PD-1 blockers, routine monitoring for adrenal dysfunction is controversial. Some authors suggest measuring morning cortisol ± ACTH in patients receiving ipilimumab-based treatment at every cycle (39), while others suggest performing a Synacthen (cosyntropin, 1–24 ACTH) stimulation test when basal cortisol measurements are inconclusive, considering that results may be falsely reassuring in the early phase of ICAI, when the adrenal glands may still respond normally to the stimulation (83).

Patients with sGC-induced ICAI require continuation of GC therapy until such treatment can be tapered to physiologic doses. At that point, testing for functional recovery of the HPA axis should be performed before attempting to stop the substitutive therapy. The optimal time to test for HPA axis recovery following prolonged sGC use remains controversial due to variability of data for timelines of when that occurs. Generally, the recovery of HPA axis has been documented as quick as about four weeks after the stop of a continuous sGCs use. It would be therefore reasonable to plan assessment of the HPA axis around that time and then every one-two months until complete recovery is documented (84). In the meantime, patients are recommended to continue adequate hydrocortisone replacement therapy (80).

Although there are no strong recommendations on how to perform an adequate follow-up, all authors agree on the importance of educating specialists other than endocrinologists and patients at risk of AI to recognize any suggesting symptom in order to obtain an early diagnosis and prompt treatment.

To conclude, it seems useful to periodically assess the presence of ICAI in particular for transient or ambiguous etiologies. In fact, improper replacement therapy leads to inappropriate GC exposure that may have a bearing on a patient’s metabolism. However, nowadays no recommendation is available.

## 4 Isolated central hypothyroidism

### 4.1 Definition and epidemiology

CH is a disorder characterized by defective thyroid hormone production and/or secretion, secondary to the insufficient stimulation by TSH of a healthy thyroid gland (4). In most patients, CH occurs in combination with other pituitary hormone deficiencies but occasionally it may present as an isolated deficit (ICH). This condition is mainly the result of anatomic or functional disorders of the pituitary gland (secondary hypothyroidism) or the hypothalamus (tertiary hypothyroidism) (4), although this distinction is no longer in use.

CH is a rare cause of hypothyroidism (about 1 in 1,000 patients with hypothyroidism) with a global prevalence that ranges from 1 in 20,000 to 1 in 80,000 individuals in the general population. It is reported to affect individuals of all ages and both sexes equally (85), although the presence of some X-linked pattern may suggest a male predominance. It is also likely that these data may underestimate the real incidence and prevalence. In fact, most countries perform a TSH-reflex assessment, both for paediatric and adult individuals but this strategy may contribute to missing diagnoses. All this considered, precise data regarding epidemiology of ICH are missing.

Infants and young children are usually affected by genetic and hereditary conditions, while expansive processes in the pituitary/hypothalamic region represent the most common cause of acquired ICH; however, milder genetic mutations can be the underlying cause of ICH with a delayed onset during life (4).

In general, there are different pathological mechanisms accounting for CH: impaired TSH secretion due to reduced hormonal reserve (quantitative, typical of the congenital forms), poor intrinsic biological activity of secreted TSH molecules (qualitative), or both (more common in the acquired cases) (85–87).

### 4.2 When to suspect an isolated central hypothyroidism and possible confounding factors

Patients with ICH may have a very heterogeneous clinical presentation. Indeed, it is related to several factors such as the etiology of the disorder (congenital ICH is generally more severe than acquired), and the patient’s age at the onset of the disease (85). Signs and symptoms, if any, may be due to the underlying hormonal disturbance and/or the possible mass effect in the case of space-occupying lesions. In the first scenario the clinical picture is usually similar to primary hypothyroidism but typically milder; this includes cold intolerance, asthenia and lethargy, constipation, bradycardia, weight gain, hoarseness of voice, thinning of hair and dry skin (85–88). Conversely, goitre is rarely present, since it is secondary to the trophic action of TSH on the thyrocytes, which in the presence of CH results, by definition, blunted. In the second case, headache, dizziness, or visual field defects are the most frequently encountered alterations (85).

Nonetheless, when a clear clinical picture is missing, people usually refer to an endocrinologist after performing routine biochemical evaluations with their general practitioner: they typically show a low-normal TSH and a low fT4 (free thyroxine); fT3 (free triiodothyronine), conversely, is usually normal in mild and moderate forms (85). Moreover, there may be several cases which go undetected since the general screening for thyroid disease is based on a cascade mechanism that starts from the detection of an altered TSH (the so-called TSH-reflex strategy). Diagnosis may be further complicated when fT4 concentration is around the lower limit of the reference range (by definition, a reference range usually comprises only 95% of a reference population and so 2.5% of healthy individuals have an fT4 below the reference interval) (89). Similarly, euthyroid patients can have up to 10% variation in fT4 levels, which is still considered normal (90). Consequently, in patients followed-up for hypothalamic-pituitary disease, a decrease in circulating fT4 levels of more than 20%, even if the values are still within the normal range, has been proposed as suggestive of CH (4, 85, 91).

Generally, it is always essential to confirm this biochemical finding on two occasions before proceeding with further analyses and with an MRI of the hypothalamic-pituitary region.

Dynamic tests using the TRH stimulation (92, 93) were more commonly used in the past but they have shown some utility even today especially in doubtful cases in which TSH values are not markedly reduced and fT4 is in the medium-low range of normality (94).

For the diagnosis of CH, it is important to exclude other confounding factors such as iodine deficiency (95) (iodine supplementation before retesting may be useful) and interfering drugs (96). Indeed, many drugs, such as bexarotene (97), mitotane (98) and immune checkpoint inhibitors (99) may alter the thyrotropic cell activity of TSH production or release determining, in general, permanent CH (96). Other drugs, like glucocorticoids, dopamine/dopamine-agonists, somatostatin analogues, metformin (100) as well as some antipsychotic and antidepressant medications (101), may exert a mild suppressive effect on TSH release without appreciably affecting circulating fT4 levels and hence without a real clinical significance (96). Finally, certain antiepileptic medications, including carbamazepine, oxcarbamazepine, valproic acid and phenytoin along with salsalate may determine falsely low levels of fT4 (secondary to pre-analytical variability due to sample dilution) and normal TSH levels in clinically euthyroid patients, mimicking the biochemical picture of ICH (96, 102, 103).

To conclude, during severe intercurrent events (organic as well as psychiatric), physiological adaptation mechanisms of the hypothalamus-pituitary-thyroid axis are involved, which can determine a biochemical picture comparable to that of ICH (this condition is called non-thyroidal illness syndrome or euthyroid sick syndrome) (104). In consideration of a reduced peripheral deiodinase activity (105), the latter condition differs from ICH because the values of fT3 are generally reduced, but a re-evaluation once the acute picture is overcome may be useful in dubious cases.

Once that CH has been diagnosed, other concomitant pituitary deficits must be assessed; in particular, it is always mandatory to exclude possible concomitant CAI that, if present, must be treated in advance to avoid triggering an adrenal crisis.

#### 4.2.1 Hereditary and congenital forms and role of genetic analysis

As of today, five genes related to ICH have been identified (89): *TRHR* (TRH receptor) and *TSHB* with an autosomal recessive inheritance and the more recently identified *IGSF1*, *TBL1X* and *IRS4* with an X-linked pathway transmission (**Table 2**).

**Table 2:** Genetic etiologies in Isolated Central Hypothyroidism.

GENETIC CONGENITAL HYPOTHYROIDISM		
Gene	Characteristics	Biochemical assessment
*TRHR*	- Autosomal recessive inheritance - Described in a few families both in males and females - Mild to moderate hypothyroidism usually asymptomatic until puberty (growth retardation)	↔ or rarely ↑ TSH levels in affected individuals ↑ TSH levels in carriers
*TSHB*	- Autosomal recessive inheritance - Most frequent cause of inheritable ICH that affects both males and females - Severe hypothyroidism with precocious onset	↔ or ↓ TSH levels in affected individuals ↑↑ α-subunit
*IGSF1*	- X-linked inheritance - Mild to moderate hypothyroidism associated to macroorchidism, GH deficiency in childhood and increased GH secretion in male adults with acromegaloid features - Delayed menarche and increased BMI in female carriers	↔ TSH levels in affected individuals ↔ or ↓ fT4 levels in female carriers
*TBL1X*	- X-linked inheritance with incomplete penetrance - Mild to moderate hypothyroidism in males associated to hearing loss - Mild hypothyroidism to euthyroidism in female carriers	↔ TSH levels in affected individuals ↔ or ↓ fT4 levels in female carriers
*IRS4*	- X-linked inheritance - Mild hypothyroidism	↔ or rarely ↑ TSH levels in affected individuals ↔ or ↓ fT4 levels in female carriers

↔ normal; ↑ increased; ↓ reduced; ICH, Isolated central hypothyroidism; TSH, Thyroid stimulating hormone; fT4, free thyroxine; GH, Growth hormone; BMI, Body Mass Index.

Mutations in *TSHB*, which encodes for the β subunit of TSH (TSHβ), constitute the most frequent cause of inheritable ICH (85, 106–108). Typically, the genetic forms manifest at birth or in the first weeks of life but mutations in the TRHR gene for example, described so far only in a few families, can determine a completely asymptomatic phenotype with only growth retardation found during puberty. Congenital hypothyroidism is one of the pathologies for which there is a newborn screening (NBS) program in many countries of the world. Unfortunately, as the main objective of these programs is the detection of the much more common condition of primary hypothyroidism (approximately only 1 in 30-40 newborns with congenital hypothyroidism has CH) (109), the vast majority of NBS are TSH-based with a cascade mechanism of dosing fT4 that starts from the detection of an altered TSH. For this reason, as often TSH may not be frankly reduced, many cases of congenital CH may go undetected and recognized only later in life resulting in possible ICH of the adulthood (109).

According to the European Guidelines, it is recommended to perform a genetic analysis in congenital cases and in cases of CH onset at any age in childhood or when the etiology is otherwise unexplained (4). Furthermore, a genetic analysis is useful as a confirmation test for conditions where fT4 may be in the low quartile of normality range interval, such as rare cases of idiopathic mild CH.

The operative method to carry out such analysis is to directly sequence a panel of candidate genes, chosen on the basis of the patient’s phenotype. In the case that this test is negative, the evaluation can be expanded to the whole genome. After the successful identification of a causative mutation related to the phenotype in the index case, the genetic analyses should be subsequently extended to all the first-degree relatives of the patient for the purposes of early diagnosis or to detect any healthy carrier of the mutation (4).

### 4.3 Therapy and follow-up

Treatment of ICH aims to restore and maintain euthyroidism and levothyroxine (LT4) is the mainstay of pharmacological therapy, since there is no evidence for the use of a combined treatment with liothyronine which is burdened, conversely, by a higher risk of overtreatment (4, 85, 88).

The replacement dose of LT4 in ICH is approximately 1.6 µg/kg of bodyweight/day, similar to primary hypothyroidism replacement therapy (110). Appropriate LT4 dose varies depending on sex (higher in women), age (lower after menopause) and concomitant treatment (such as oestrogens); similarly, younger people often require higher dosages (111). Children and young adults can generally start a full replacement dose of LT4 when commencing the treatment, while in the elderly it may be safer to start with low doses (e.g., 25-50 µg/day) to increase with caution (4).

As of today, the proper assessment of the adequacy of LT4 therapy in patients with CH and subsequently for ICH still remains an object of debate. Indeed, TSH serum levels are not fully reliable to monitor replacement therapy like in primary hypothyroidism, because they appear to be suppressed even during low-dose LT4 treatment. All this considered, it is safe to ascertain that TSH levels above 0.5-1.0 mU/L should be considered as a sign of insufficient replacement in such class of patients (4, 85). Over the last years, increasing evidence investigating the efficacy of LT4 replacement therapy shows that patients with CH have significantly lower levels of fT4 with respect to patients with primary hypothyroidism adequately treated; therefore, subjects with CH are often left untreated, thus increasing their long-life cardiovascular risk (90, 110). For these reasons, in the context of ICH, the best markers to properly set the adequate therapy are fT4 and fT3, aiming at bringing fT4 values in the upper range of normality and fT3 in the normal range of population (4, 109).

The first evaluation after starting the treatment should occur after 6-8 weeks and the measurement of fT4 should anticipate the assumption of LT4 therapy. Once reaching the aforementioned biochemical target and obtaining the concomitant resolution of the symptoms, if present, thyroid function can be checked periodically, at least annually (4, 90).

## 5 Isolated hypogonadotropic hypogonadism

### 5.1 Definition and epidemiology

Isolated hypogonadotropic hypogonadism (IHH) is defined as a condition of secondary hypogonadism not associated with other pituitary deficits. It can manifest as a congenital (CIHH) or acquired (AIHH) disruption of the hypothalamic-pituitary-gonadal (HPG) axis (112). Despite lack of definite data, IHH can be considered an infrequent condition as a whole: for CIHH, a male predominance is reported (2-5 males to 1 female) with an estimated prevalence between 1/10,000 and 1/86,000 in males, while no piece of information is available about CIHH in females (113). Furthermore, AIHH has still no clear evidence about incidence or prevalence, probably due to the wide number of involved etiologies and due to the variability of the definition of this condition between studies and over time.

CIHH is mostly related to a genetic alteration and among 50% to 60% of the patients have an associated olfactory dysfunction (anosmia or hyposmia), defining Kallmann syndrome (114).

AIHH presents when a disruption of the hypothalamus or the hypophysis alters the release of gonadotropin-releasing hormone (GnRH) or of LH and FSH, leaving untouched the release of the other pituitary hormones.

The clinical signs can be present early during life, from childhood and in particular puberty, when IHH can manifest with delayed or absent pubertal development until clinical pictures characterised by infertility and hypogonadism in adulthood. Therapies are available to accomplish an eugonadal state and, whether possible, fertility.

After this glimpse, we will further describe these sections hereafter.

#### 5.1.1 Etiopathogenesis

The etiology of IHH is mostly related to the time of onset of the clinical picture. Talking about CIHH, this condition is mainly linked to genetic causes, thus familial history can insinuate the doubt. The primarily involved genes are associated with GnRH, which regulates the release of LH and FSH by the pituitary gland. The majority of hypothalamic GnRH neurons originates in the olfactory placode around the 5th gestational week and migrates alongside the olfactory, vomeronasal and terminal nerves until arrives at mediobasal hypothalamus, infundibulum and periventricular region (115). CIHH is a result of the failure of GnRH neurons to differentiate, develop or function properly or, in a subgroup of patients, there may be a GnRH resistance in pituitary gonadotropic cells (e.g., loss of function mutations in the GnRH receptor) (114). The olfactory defects can occur as a result of the close link between GnRH neurons and olfactory axons; in fact, an embryonal abnormal migration of GnRH neurons and olfactory fibres from their origin in the olfactory placode to the forebrain can result in a hypoplasia or aplasia of the olfactory tract/bulbs associated to GnRH deficiency, that is usually described in CIHH as Kallmann Syndrome (116).

Many genes have been pointed out in the pathogenesis, including X-linked recessive (such as *ANOS1*, formerly known as *KAL1*), autosomal dominant (e.g., *FGFR1*), and autosomal recessive genes (like *KISS1-KISS1R, GNRHR*); even oligogenic (caused by more than one gene defect) and sporadic forms have been described (114). Mutations in *ANOS1* occur in approximately 4.5% of all CIHH patients (117–122), with an important olfactory and reproductive phenotype. *FGFR1* mutations are the most commonly known molecular cause of CIHH that may cause isolated defects in GnRH neuronal proliferation and migration without necessarily affecting olfactory bulbs and function (123, 124). Kisspeptin produced by periventricular and arcuate nuclei, is a pivotal regulator of GnRH neurons. Mutations in Kisspeptin, encoded by the *KISS1* gene, or of its receptor encoded by *KISS1R* and expressed in the surface of the GnRH secreting neurons, are linked with patients with normosmic CIHH (125). Eventually, GnRH receptor (*GNRHR*) mutations have been shown to be responsible for a significant proportion of normosmic CIHH cases, associated with a broad phenotypic reproductive spectrum, varying from partial to complete GnRH resistance (126–128).

AIHH is related to defects of the hypothalamus or of the pituitary gland, such as trauma, inflammation, infiltrative diseases, and tumors (**Table 1**) (129). These conditions can manifest both in early and adult life. A certain percentage of AIHH may present with a genetic alteration, that seems to be associated with a mild pattern of pubertal delay, suggesting a possible underlying pre-existing slight impairment of HPG axis (117, 130, 131); moreover, a part of AIHH patients present characteristics of classical CIHH of pre-pubertal onset, including anomalous olfactory bulbs and sulci on MRI (132).

### 5.2 When to suspect an isolated hypogonadotropic hypogonadism

Clinical pictures can be heterogeneous, ranging from clear manifestations in newborn (such as cryptorchidism and micropenis in males, while in females there are not clear signs) to nuanced manifestations in adults. Moreover, some genetic defects may manifest with alterations in other organs such as olfactory deficit, cleft lip or palate, dental agenesis, ear anomalies, congenital hearing loss, renal agenesis, mirror movements (bimanual synkinesis) or skeletal anomalies. Moreover, acquired conditions can be associated with other symptoms, related to the causing condition (132–134).

*Puberty development*: puberty is a pivotal phase where suspicions for IHH can arise, in particular for CIHH: there can be no puberty or just the first signs, with a partial development. Girls may have an initial breast development associated with primary amenorrhea (only a few shows a couple of menstrual bleedings) associated with a variable pubarche; boys usually present no (the majority, around ⅔) or only initial testicular development (testicular size > 4 mL, a milestone of male puberty) with no further increase. Eunuchoid proportions (arm spans exceeding by 5 cm the height) are a typical presentation, due to delayed epiphyseal closure (116).

*Adult*: in adult life, men with IHH can present with signs and symptoms of hypogonadism such as loss of libido, erectile dysfunction, loss of body hair growth, fatigue, reduced bone mass, muscles hypotrophy, low mood. Normal sized genitalia (penis and testicles with a normal or slightly reduced volume), a normal stature and a low-pitched voice should be present, due to the normal pubertal development and androgenization that occurred (112). Women can complain of secondary oligo-amenorrhea up to amenorrhea, flushing, fatigue, loss of libido, reduced bone mass, low mood. In these patients, the presence of hyperandrogenism (such as acne, alopecia, hirsutism, clitoromegaly) should be assessed, to exclude androgen-excess related amenorrhea (135).

When CIHH is suspected, clinicians should investigate the sense of smell in order to find hypo-anosmia (113). On the other hand, if an AIHH is suspected, it is useful to investigate conditions that may impair HPG axis, asking about e.g., headache, visual impairment, breast tenderness and discharge.

### 5.3 Diagnosis

In this section we are going to focus on the adolescent-adult diagnosis to remain faithful to the aim of this paper. For a more punctual reading about infant diagnosis, we suggest referring to dedicated articles. A proper anamnesis should be collected, in order to evaluate familial pattern of this condition and to collect information about general health status, including potential exposition to endocrine disrupting chemicals (136).

In puberty, a growth delay is challenging to distinguish between IHH and constitutional delay of growth (CDGP) and puberty. After a clinical evaluation, initial assessments should comprehend the evaluation of HPG axis: gonadotropins (LH, FSH) associated with total testosterone (TT), in boys and with estradiol (E2) in girls. IHH is characterised by low gonadotropins and low levels of TT or E2. When the suspicion between IHH and CDGP exists, no gold standard evaluation is recommended to dispel this uncertainty, thus a GnRH stimulation test is the one proposed, but it lacks reliability (137). Adjunctive assessments that may help in the diagnosis are inhibin B and Anti-Müllerian Hormone (AMH). Inhibin B is a glycoprotein produced by Sertoli cells and it correlates with spermatogonia function and status. Its serum levels rise with the onset of puberty and tend to be very low in patients with CIHH (138). The FSH-stimulated inhibin B has shown to correctly differentiate pubertal delay from HH; however further studies are needed to confirm this finding (139). In adults who went through puberty, the assessment of inhibin B may not be reliable to further point out IHH. In fact, these men are supposed to have developed a normal testicular volume (underlying a proper germ cell proliferation) and consequently potentially normal inhibin B levels. Moreover, AMH, a hormone released by Sertoli cells and granulosa cells, could be a useful tool in pre-pubertal boys. In fact, in CIHH boys serum AMH tends to remain elevated for age (due to lack of down regulation mediated by testosterone rising), but lower than expected for the patient’s Tanner stage (due to the lack of FSH feedback that induces an elevation of AMH) (140). In adults, if the clinical picture is consistent with IHH, serum LH, FSH associated with TT, sex-hormone-binding globulin (SHBG), and albumin to evaluate calculated free testosterone (CFT) in men, and associated with E2 in women should be evaluated. CFT is a particularly useful assessment in those conditions that may raise SHBG (e.g., liver dysfunction, obesity, HIV) and in which the evaluation of lone TT may improperly diagnose hypogonadism. Sense of smell assessed by a formal smell test should be addressed to help in the diagnosis (141).

Through an extensive anamnesis, it is important to exclude functional hypogonadism in these men; this is a potentially reversible condition, with a hormonal pattern similar to IHH but with no link with a direct disruption of HPG axis. It is rather related to an impairment of general health condition (diabetes, metabolic syndrome, multiple comorbidities, acute disease, sleep apnoea, HIV, and energy imbalance that can result from a strenuous physical activity e.g., endurance sports) or drug induced (opioids, corticosteroids, androgenic/anabolic androgenic therapies, GnRH analogues, psychotropic treatments) (129, 142). Notably, the abuse of anabolic-androgenic steroids has been growing exponentially among elite and amateur athletes, of both genders (143). The use of aromatizable steroids, the imbalance between testosterone and estradiol serum levels, and their withdrawal after long-term use seem to represent the main reasons for men to incur an anabolic steroid-induced hypogonadism (ASIH) (144), which may not properly recover over time (129).

In women presenting with amenorrhea, pregnancy must be firstly excluded by evaluating serum β-hCG (human chorionic gonadotropin). Then, hormonal levels should be evaluated after a progesterone challenge test (e.g., using a medroxyprogesterone acetate for 10 days and after the withdrawal of the drug check for menstrual bleeding), within a week from the start of the bleeding, in order to proper interpret the assessment and to exclude other conditions that may mime a secondary hypogonadism. If signs of clinical hyperandrogenism are present, it is useful to assess serum androgens (mainly TT) and evaluation of free androgen index (FAI) using SHBG (145), but also 17-hydroxyprogesterone (17-OHP) and dehydroepiandrosterone sulphate (DHEA-s) could be useful to exclude other conditions (such as polycystic ovary syndrome) that may elicit this menstrual pattern. Adjunctive evaluations should exclude hyperprolactinemia, severe hypothyroidism, and the presence of functional amenorrhea (a form of chronic anovulation that is not due to identifiable organic cause and usually related to excessive energy expenditure). A gynaecological evaluation should be performed especially in younger women (135). Adjunctive assessments, such as AMH, are unfortunately not useful to further address the diagnosis, yet. In fact, in females with IHH, AMH can be either low, normal, or high (146).

After assessing HH, a second blood test should be performed to confirm the results. Then, it is appropriate to evaluate the whole anterior pituitary function to exclude other associated abnormalities and to properly diagnose IHH. Eventually, an MRI should be performed to evaluate the hypothalamic-pituitary area, the olfactory bulbs, and to investigate the possible etiologies of AIHH above mentioned. A formal smell test can be ordered to find alterations (113). It is interesting to highlight that even some AIHH patients also show alterations of olfactory bulbs on MRI that are commonly associated with classical CIHH (132).

When the diagnosis has been made, a genetic counselling should be offered accordingly. The current increased use of next generation sequencing (targeted exome) in clinical practice allows the identification of causative genes without necessarily completing an exhaustive search for associated signs (147).

### 5.4 Therapy and follow-up

Once IHH has been diagnosed, a proper treatment, in accordance with the age of the person, should be offered. During childhood, an early diagnosis can provide better outcomes in terms of puberty development, to best benefit for sexual, bone and metabolic health, and help minimize some of the possible psychological effects (116). In general, in CIHH the proper age to start puberty treatments should be individualised and the aim is to obtain a final endocrine environment with gradual increase of doses to mimic physiological pubertal development (148). In boys there are two main therapeutic approaches: induction of virilization by testosterone injection (which has more efficacy and a more robust body of literature) or transdermal gel (it could be more physiological but scarce data are available) in order to reduce sexual infantilism and psychological distress (low doses if started at about 12 years of age and subsequently titrated or higher doses if started in later adolescence or early adulthood) (148). Another possible approach is the use of pulsatile GnRH or gonadotrophin therapy (hCG ± FSH) resulting in an endogenous increase of serum TT (even intratesticular) that results in improving testicular growth. This approach seems to grant better responses of sperm retrieval, in particular in men with cryptorchidism that have poorer fertility prognosis (116). A growing body of evidence suggests that the combination therapy with recombinant FSH, especially before adding hCG, is significantly more effective than hCG alone both for inducing spermatogenesis and increasing testicular volume (117, 149–151). After pubertal induction, boys can continue these treatments or switch to testosterone replacement therapy, which can be made of mild or long-term intramuscular injections, transdermal gel or patches, tablets, intranasal and subcutaneous implants (**Table 3**). When men desire fertility, they stop assuming testosterone and can start a treatment with gonadotropins (hCG + FSH) or pulsatile GnRH therapy in order to induce spermatogenesis (113).

**Table 3:** Summary of treatment options in male adult affected by Isolated Hypogonadotropic Hypogonadism.

ADULT MALE HYPOGONADISM			PROs	CONTRAs
Testosterone enanthate, cypionate or mixture of esters	150-250 mg IM every 2-4 weeks	Titrating dose based on clinical signs and symptoms, serum testosterone levels	Self-injection Easily available	Higher risk of erythrocytosis Frequent serum testosterone peaks Frequent injections
Testosterone undecanoate	750-1000 mg every 10-14 weeks		Longer interval injections Stable serum testosterone levels	injection by a health care provider risk of pulmonary oil microembolism
Testosterone gel	40 mg-80 mg/daily		Easy avoidable side effects Non invasive Mimics physiology	Daily administration Skin irritation Possible skin to skin transfer of therapy
Testosterone patch	2.5–5 mg/day		Mimics physiology	Skin irritation, Possible issues with frequent showering or certain lifestyle
Oral testosterone	Undecanoate testosterone 158–396 mg twice daily		Oral administration	Daily multiple doses; need lipid rich meals; gastrointestinal side effects; hypertension
Intranasal testosterone	11 mg twice/die		Easy to administer	Sense of taste alteration
Testosterone pellets	75 mg pellets, 3-4 every 4-6 months		Easier compliance	Risk of local side effects (extrusion, fibrosis, infection) Higher cost
ADULT SPERMATOGENESIS INDUCTION				
Gonadotropins	Starting dose: hCG 500 UI SC thrice/week + FSH 75-150 UI thrice/week	Titrating dose: - hCG increase based on serum testosterone - FSH increase based on serum FSH and sperm count	Self-injections	Require optimal compliance Need frequent injections
Pulsatile GnRH	SC pump: 25 ng/kg per pulse every 120 min	Dose e adapted based on serum testosterone levels	Most physiological	Not easily available Pituitary resistance (rare)

Adapted from Young et al., 2019 and Nordenstrom et al., 2022.

SC, subcutaneous; IM, intramuscular; hCG, Human chorionic gonadotropin; FSH, Follicle stimulating hormone.

Data about puberty induction in girls with IHH are scanty. An incremental dose, by 11-13 years of age, is recommended over a period of 2–3 years to reach a proper adult dose (148). This slow progression is pivotal to avoid a negative impact on breast development or growth (152). If the diagnosis is made in late adolescence, clinicians may start with higher doses. Bioidentical human estrogens (estradiol/17β-estradiol) are the preferred formulations that can be used orally or transdermally. The metabolic effects of transdermal and oral routes did not show differences in fibrinogen and antithrombin activity, glucose and insulin, liver enzymes activity, lipids concentration, plasma renin, as well as insulin growth factor 1 (IGF-I) levels (153). After at least 2 years of treatment with estradiol for puberty induction, progesterone should be started after breakthrough bleeding, to avoid the risk of endometrial hyperplasia (148). No preferred routes are currently known, and progesterone can be delivered orally, vaginally, transdermally, intranasally or intramuscularly, with a 10-day cycling treatment. After about 3 years of pubertal induction, girls should have reached adult doses of estrogen therapy (transdermal or oral routes are suggested) always associated with a cyclic therapy with progesterone (**Table 4**). Unlike boys, there is no specific data about the need of gonadotropin therapy during adolescence in girls. However, when the fertility desire comes, the available treatment is the use of exogenous gonadotropins, and clinicians should modulate hCG and FSH resembling the various phases of the menstrual cycle (**Table 4**). To induce fertility, gonadotropin therapy is effective, but is much more likely to be complicated by multiple pregnancy and ovarian hyperstimulation due to lack of the protective HPG feedback that normally would lead to selection of the dominant follicle (154). Women with secondary hypogonadism seem to have a very narrow gonadotropin therapeutic window between low and excessive response, with a potential high number of responsive follicles (146).

**Table 4:** Summary of treatment options in female adult affected by Isolated Hypogonadotropic Hypogonadism.

ADULT FEMALE HYPOGONADISM			PROS	CONTRAS
Estrogenic therapy (patch)	50-100 micrograms/24 h Applied twice/week	Titrating dose based on clinical signs and symptoms	No first passage effect	Skin irritation, Possible issues with frequent showering or certain lifestyle
Estrogenic therapy (gel)	Estradiol or estradiol hemihydrate 0,5 to 2 mg/die		No first passage effect	Skin irritation; need to be accurately dried
Estrogenic therapy (tablets)	Micronized or valerate estradiol 1-4 mg/die			First passage effect
Progesterone	e.g., Micronized progesterone (100-200 mg/die for last 10 days/month) vaginal route			
ADULT OVULATION INDUCTION				
Gonadotropins	*Follicular Phase:* FSH + LH) 75 to 150 IU SC daily, *Ovulation phase:* induced by hCG 6500 IU *Luteal phase:* hCG 1500 UI every 3 days, thrice or progesterone 200 mg intravaginally daily	Follicular phase: depending on follicular growth (serum estradiol and ultrasonography)	Self-injection	Higher risk of overstimulation and multiple pregnancies
Pulsatile GnRH	SC pump: 15 mg per pulse every 90 min	Dose adapted based on response, up to 30 mg per pulse	Less risk in multiple pregnancy; most physiological treatment	Pituitary resistance (rare)

Adapted from Young et al., 2019 (113) and Nordenstrom et al., 2022 (148).

SC, subcutaneous; hCG, Human chorionic gonadotropin; FSH, Follicle stimulating hormone; LH, luteinizing hormone.

IHH, in particular CIHH, has been traditionally considered a permanent condition. Patients typically require lifelong treatment and monitoring in order to maintain sexual function, fertility and secondary sexual characteristics, although 5-20% of male patients (3, 149, 155–158) exhibit a spontaneous recovery (permanent or transient) of gonadal function. Moreover, some men seem to sustain reversal of the disease after discontinuation of hormonal therapy (no clear connection with genetic defects or clinical presentations are currently known). Reversal should be suspected if testicular volume increases during testosterone administration or in cases of spontaneous fertility in IHH patients (114). Conversely, there are no cases of documented reversal of IHH in women. It is reasonable to think that, in the case of AIHH, when the subsequent cause has been underlined and treated, hypothalamic-pituitary function may be restored. Progressively lowering hormone replacement therapy until withdrawing it for a certain period, could be a useful approach to evaluate symptoms and signs reported by the patient and to perform hormonal assessment to definitively ascertain HPG recovery.

## 6 Isolated growth hormone deficiency

### 6.1 Definition and epidemiology

GHD results from a decrease in GH secretion by the pituitary gland, leading to a reduction of IGF-I. Although GHD usually represents the first pituitary deficit to appear in combination with others, rarely it may present as an isolated deficit (159).

Isolated GHD (IGHD) is widely studied in infants and childhood population as a result of genetic disorders and anatomical abnormalities. In these cases, GHD syndrome, clinically characterized by short length, recurrent hypoglycemia, and severe dwarfism, is easily suspected; in adulthood instead, the lack of pathognomonic signs and the overlap with other clinical conditions, makes the diagnosis more challenging. However, the interest on GHD diagnosis in adulthood has grown during the last decades thanks to the availability of different dynamic tests and positive data of rhGH (recombinant human growth hormone) treatment.

Incidence and prevalence of adult-onset GHD (AO-GHD) are difficult to estimate. Sassolas G et al. in 1999 conducted an epidemiological study to evaluate the frequency of this syndrome in the French population; they analyzed data from 1652 patients with a history of hypothalamic-pituitary damage and they found an incidence of 12 GHD per million of adults and a prevalence of 46 per million (160). Another nation-wide cohort study in Denmark reported an incidence of 1 per 100.000 people yearly and 2 per 100.000 when childhood onset GHD (CO-GHD) were included, with approximately 15-20% of cases being transition of CO-GHD into adulthood (161, 162). Combining both AO-GHD and CO-GHD yields an overall prevalence of 2 to 3 per 10.000 population (163). Specific epidemiological data of isolated GHD, however, are not available.

### 6.2 Etiopathogenesis

About 15-20% of cases of adult IGHD are transitions of CO-GHD (161). In this context, the most common cause is idiopathic deficit followed by genetic syndromes (**Table 5**).

**Table 5:** Genetic etiologies and their clinical and biochemical phenotype in Isolated Growth Hormone Deficiency (164).

GENETIC GROWTH HORMONE DEFICIENCIES			
Inheritance	Type	Gene	Phenotype
Autosomal recessive	IA	*GH1*	↓↓ stature ∅ serum GH + anti-GH antibodies on treatment*
Autosomal recessive	IB	*GH1* *GHRH*	↓ stature ↓↓ serum GH ∅ anti-GH antibodies on treatment
Autosomal dominant	II	*GH1*	↓↔ stature - normal or hypoplastic anterior pituitary on MRI scan - Other pituitary hormone deficiencies
X-linked	III	*SOX3* Other	↓ stature ↓ serum GH agammaglobulinemia ± intellectual disability and ectopic posterior pituitary on MRI scan

↔ normal; ↑ increased; ↓ reduced; ∅ undetectable; + detectable; ± possible; GH, growth hormone; MRI, Magnetic Resonance Imaging.

*The initial good response to exogenous GH is hampered by the development of anti-GH-antibodies (165) (166).

Childhood idiopathic IGHD is a well-recognized form, characterized by growth failure due to the lack of GH action in absence of both organic lesions and genetic mutations (167). Still, the majority of these patients undergo subsequent recovery of the somatotropic axis during transitional age (168, 169), resulting in only a small fraction of them still having GHD in adulthood. The definition of idiopathic GHD in adulthood, therefore, remains controversial: Melmed described it as a rare condition in which rigorous criteria must be applied, excluding all known common organic causes (170).

Conversely, when there are specifically genetic mutations responsible for GHD, patients CO-GHD remain always adult IGHD. A mutation responsible for the condition has been identified in up to 11% of isolated CO-GHD; *GH1, GHRH, SOX3* are the most studied genes with four types of genetic forms recognized (IA autosomal recessive, IB autosomal recessive, II autosomal dominant, III X-linked) (159).

As previously pointed out, GH-axis is the most vulnerable axis to pathological insult to the pituitary gland; therefore, GHD can be the first detectable deficit (171, 172).

Apart from known genetic syndromes, identifying IGHD in adults is not easy because the majority of studies does not differentiate between GHD in combination with other pituitary deficits (multiple pituitary hormone deficiency, MPHD) from isolated forms. To our knowledge, only one study focused on the comparison between IGHD and MPHD in adults. In this study, a sub-analysis of KIMS database (Pfizer International Growth Study Database) conducted by Roger Abs et al. (166), no significant difference regarding causes was found, and hypothalamic-pituitary tumors and/or their treatment regimens constitute the most frequent causes for both groups IGHD and MPHD. Despite not being statistically significant, it was observed a higher rate of other sellar tumors (germ cell tumor, hamartoma, chordoma, glioma, meningioma, cyst) in IGHD (166). While macroadenomas are more likely associated with MPHD (165), small pituitary lesions, in particular non-functioning adenomas, may present with only IGHD (42).

Moreover, partial or complete hypopituitarism is often a consequence of pituitary surgery, but unlike other pituitary deficits that may recover over time, the somatotropic axis represents the one with the lowest probability to be reacquired (173).

Bearing in mind the consequences of the treatment of pituitary lesions, radiotherapy plays an important role in the onset of IGHD. As previously pointed out, GH-axis cells seem to be more radiosensitive, with damage tending to be irreversible even at very low doses (174). Specifically, in adults, the risk of GHD is dose and time related and continues to increase during time with a median onset latency of 27 months (175).

While doses of up to 18 Gy result in a rapid onset of IGHD, lower doses (≤ 10 Gy) could cause a late-onset damage, with a cumulative risk that increases with longer follow-up (176, 177), making therefore important investigating previous history of RT in childhood, both for sellar and non sellar tumors (178).

IGHD is also related to TBI (even mild) and SAH with a frequency at 3 months from the event that can be up to the 30% of the cases; anyway, in these situations IGHD is frequently transient, with a complete axis recovery by 12 months (22, 179, 180). An interesting review conducted by Gasco et al. has recently analyzed the literature data on hypopituitarism and GHD related to TBI. The pathophysiological mechanisms involved include neurotransmitter-mediated excitotoxicity, secondary ischemia and inflammatory response. Particularly, long hypophyseal portal vessels represent the only supply to the lateral portion of the gland and to the pars tuberalis, mostly populated by GH, PRL and FSH/LH secreting cells, subjected to a higher risk of damage. GHD is common both in the acute stage of TBI (first two weeks) and in the chronic phase (3 months after TBI) with a severe impact on the rehabilitation post-TBI (181).

A peculiar cause of TBI-induced pituitary dysfunction is represented by sport injuries. It was observed that amateur boxing and kickboxing, as a consequence of chronic and repetitive head trauma, may both cause IGHD (182, 183). Nervous system infections (25, 184, 185) and ischemic stroke (186–188) are other rarely reported causes of GHD. Anyway, these data are derived from broader studies on hypopituitarism and are not specific to IGHD.

Finally, GHD may be the possible consequence of ICPi but data on the isolated form are not available; as a matter of fact, in this clinical context somatotropic axis is surely less studied because potential substitutive therapy would be contraindicated from the oncological point of view (99).

### 6.3 When to suspect an isolated growth hormone deficiency

Nowadays, GHD is a well-recognized syndrome characterized by increased fat body mass and decreased lean one, osteoporosis and augmented fracture risk, hypertension, abdominal obesity, diabetes mellitus (DM), dyslipidemia and enhanced thrombotic factors with an increased global cardiovascular risk. Reduced vitality, muscle strength and early exhaustion are the most reported symptoms (189). Rogers et Arm (166) showed no statistically significant differences in clinical presentation between IGHD and MPHD, supporting the thesis that GHD alone is responsible for all metabolic aspects. Because of the high frequency of these signs and symptoms in the general population, clinicians are usually not able to clinically suspect IGHD. This aspect explains why GH-stimulation tests are generally performed only in patients with a suggestive clinical context and with a history of possible pituitary damage.

In the presence of documented genetic alteration, re-testing in transition age is unnecessary. In idiopathic CO-GHD, conversely, patients with low-normal (between 0 to -2DS) or low (< -2DS) serum IGF-I levels, re-testing after at least 1 month withdrawal of rhGH is mandatory (159).

Recent guidelines have focused on the need to test for GHD only patients who may actually be treated if the biochemical diagnosis is confirmed (159). The Food and Drug Administration approval of rhGH replacement therapy for adults lists active malignancy as a contraindication, considering the known growth-promoting effects of GH and IGF-I. Several studies demonstrated the safety of rhGH on tumor regrowth or recurrence after surgery in patients with pituitary tumors or craniopharingiomas making IGHD diagnosis still recommended in these cases (190–197).

IGHD should definitely be suspected in patients with previous history of TBI or SAH because of the high frequency of the condition in this setting, and considering the positive effect of therapy on the reduction of post-TBI sequelae and subsequent rehabilitation (181). Indications for testing are: moderate/severe TBI based on GCS score or mild complicated TBI (i.e., those who need hospitalisation, neurosurgical intervention, monitoring in Intensive Care Units or present anatomical changes on computed tomography scan, always on a risk/benefit ratio (181).

### 6.4 Diagnosis

GH secretion is pulsatile; thus, random GH levels have no diagnostic value in the evaluation of GHD. The measurement of serum IGF-I alone does not allow to properly identify GHD; indeed, many physiological and pathological conditions, such as older age, DM, malnutrition, chronic liver disease and renal failure may lower serum IGF-I levels in adults, making the definition of a threshold challenging. Moreover, it is possible to assess normal IGF-I levels even in patients with GHD diagnosis. For example, an interesting study conducted by Yuen *at al* (159). has shown that patients with clinically non-functioning microadenomas may have normal serum IGF-I but biochemical evidence of incomplete response to GHRH-arginine test. For all these reasons, in case of strong suspicion of GHD, a stimulation test (ITT test, GHRH+arginine test, glucagon stimulation test, macimorelin stimulation test) is mandatory to confirm the diagnosis.

Furthermore, several studies have demonstrated that IGF-I levels are significantly higher in IGHD than in MPHD patients; similarly, these patients show a tendency for a higher GH response to stimulation tests than people with MPHD (166, 198). Again, a strong negative correlation between baseline IGF-I and the number of additional hormone deficiencies has been reported (199). Therefore, in patients with no other hormonal deficit, some authors suggest performing two tests in order to confirm the IGHD diagnosis (166, 200).

ITT still remains the gold standard. However, because of safety concerns, other tests are more widely performed in clinical practice, such as GHRH+arginine and GST. Furthermore, the identification of GH cut-offs is a widely debated issue because of the variability related to factors such as body mass index (BMI) and age. Diagnostic cut-offs for GHD generally recognized for the various possible tests are summarized in **Table 6** (159, 201). As of today, regarding ITT, the diagnostic criteria do not correlate with BMI or age. A first attempt to integrate these data has been performed by Gasco et al, who have identified through ROC curves the best cut-off BMI-related to avoid false positive results (≤ 3.5 μg/L if BMI<25 Kg/m^2^, ≤ 1.3 μg/L if BMI≥25 Kg/m^2^) (202).

**Table 6:** Growth Hormone Deficiency diagnostic cut-offs with different tests.

TEST	GH PEAK
ITT	≤ 3-5 μg/L
Macimorelin	≤ 2.8 μg/L
GST	≤ 3.0 μg/L in normal-weight patients ≤ 3.0 μg/L in overweight patients with a high pre-test probability ≤ 1.0 μg/L in overweight patients with a low pre-test probability ≤ 1.0 μg/L in obese patients
GHRH+arginine	≤ 11.5 μg/L in normal-weight patients ≤ 8.0 μg/L in overweight patients ≤ 4.2 μg/L in obese patients

GH, Growth hormone; ITT, Insulin tolerance test; GST, Glucagon stimulation test; GHRH, Growth Hormone Releasing Hormone.

The best test and the correct timing to perform it has to be chosen taking into account the clinical history. Regarding this, no specific guidelines are available. Anyway, the evaluation of GH-axis with dynamic tests should be carried out after having properly corrected other concomitant pituitary hormone deficits.

As far as TBI is involved, it is recommended to test somatotropic axis at least six months after post-acute phase (203), even though other Authors proposed to postpone the evaluation until one year after TBI (204). Moreover, ITT is often considered unsafe in these patients because of the possible contraindications (i.e., seizure, cardiac disease). In the same way, in the post-surgery and SAH context, an early evaluation is not recommended because of the possibility of subsequent recovery over time. In patients with a previous history of radiotherapy, considering the possibility of late onset of IGHD and hypopituitarism, a longer follow-up should be scheduled, although no data regarding actual duration are available. Moreover, in previous cranial irradiation GHRH + Arginine is not recommended (190), because of the possibility of coexisting hypothalamic defect (cause of false negative outcomes).

### 6.5 Treatment and follow-up

GHD therapy is based on the hormone replacement with rhGH. Many commercial products are available and there is no evidence about the superiority of one on another. RhGH therapy has shown to be of benefit for body mass composition, skeletal integrity, lipid profile and muscle performance, although an improvement in overall cardiovascular mortality has not been demonstrated.

RhGH therapy is contraindicated in presence of an active malignancy, severe illness or advanced non-proliferative diabetic retinopathy. Individual patient characteristics should be taken into account when choosing the starting dose: 0.1 to 0.2 mg/day in patients with concurrent DM, obesity or age>60 years, 0.2-0.3 mg/day in 30-60 years, 0.4-0.5 mg/day in age < 30 years. Starting doses may be higher for patients transitioning from pediatric treatment (159). An evaluation every 1-2 months is suggested to properly titrate rhGH dose based on clinical response, side effects and individual consideration. Serum IGF-I levels and subjective perception of symptoms with validated quality of life questionnaires (QoL-AGHDA) (205) are the best markers to monitor the ongoing therapy. When maintenance rhGH doses are achieved, serum IGF-I, fasting glucose, glycosylated hemoglobin, lipidic profile, BMI, waist circumference and waist-to-hip ratio may be assessed at 6 to 12-months intervals and bone mineralization dual x-ray absorptiometry every 24 months (159).

It is important to remember that the initiation of replacement therapy can unmask the presence of other hormonal deficits but, at the same time, that GHD may simply be the first of several hormonal axes deficits to appear. It is therefore necessary to periodically monitor the remaining anterior-pituitary function. In particular, we need to consider that any clinical deterioration not otherwise explained may be related to the onset of other pituitary deficits, like CAI (69). For this reason, Guidelines suggest checking serum fT4 and the HPA axis annually, either by morning cortisol measurement or cosyntropin-stimulation test (159).

A review from Cerbone M. and Dattani M.T. focused on the risk factors for progression from IGHD to MPHD, identifying absent pituitary stalk, ectopic posterior pituitary, abnormal corpus callosum, empty sella, septo-optic dysplasia, longer duration of follow-up and genetic defects as risk factors for progression, with strong evidence (206).

No differences on therapy response were observed between MPHD and IGHD (166).

## 7 Isolated prolactin deficiency

### 7.1 Definition and epidemiology

Prolactin (PRL) deficiency is a condition characterized by low or undetectable PRL levels, as a potential consequence of the aforementioned causes of anterior pituitary dysfunction (**Table 1**). It usually presents in association with other hormonal insufficiencies and in this context, it has been reported as a marker for a more severe degree of hypopituitarism (207). Isolated PRL deficiency (IPRLD), conversely, is an extremely rare condition and up to now only few cases have been reported in literature (208–213). IPRLD is generally considered idiopathic; however, in some cases a potential genetic etiology can be speculated. In fact, in literature familial cases of hypoprolactinemia (e.g., mother and daughter) associated with puerperal alactogenesis are reported (210) and an autosomal recessive inheritance has been hypothesized (212). However, no genetic investigations were performed in the described cases and, to date, a specific gene for IPRLD has never been discovered. Finally, in one patient an autoimmune disorder selectively affecting the lactotroph cells has been outlined (212).

The most frequent cause of IPRLD is iatrogenic. Dopamine agonists (DA) are commonly used in clinical practice to treat PRL-secreting tumors and the chronic administration of these molecules (cabergoline, bromocriptine, quinagolide or pergolide) can sometimes lead to an inhibition of circulating PRL levels (214). In fact, lower PRL levels during DA are associated with long-term recovery in patients with prolactinomas (215). Even aripiprazole, an atypical antipsychotic agent with a partial agonist activity on dopamine receptors (D2), could determine a reduction and suppression of PRL levels when administered at higher doses than 5 mg/day (216).

Moreover, hypoprolactinemia has been described in patients with hemochromatosis (217) and in patients with pseudohypoparathyroidism, a rare genetic disorder characterized by a resistance to parathyroid hormone (PTH) caused by GNAS (guanine nucleotide binding protein, alpha stimulating) mutations. In pseudohypoparathyroidism lactotroph cells has shown a lack of responsivity to PTH, that normally increases plasma PRL in adults (218).

### 7.2 When to suspect an isolated prolactin deficiency and diagnosis

All reported cases of IPRLD described in literature concerned women, and the condition was revealed by a lactation failure occurring after delivery (puerperal alactogenesis). To date, in men and in non-lactating and non-pregnant women, PRL deficiency has no clinical implications, even if recent data have demonstrated an apparent impaired sexual functioning, reduced wellbeing and increased cardiometabolic risk in patients with iatrogenic IPRLD (219).

In IPRLD women, usually no alteration of menses was reported. However, some women suffered from oligomenorrhea (209, 211) while some others reported a delayed ovulation (209) and a woman reported the necessity to be treated with clomiphene citrate in order to conceive (209). Due to the paucity of data, no cause-and-effect correlation can be determined between these conditions and hypoprolactinemia.

The diagnosis of IPRLD is established in the context of a normal pituitary function with the evidence of low or undetectable PRL levels and failure to increase after administration of TRH or antidopaminergic medications (e.g., metoclopramide or chlorpromazine) (212, 220).

### 7.3 Therapy and follow up

As of today, it is still not clear how to treat women with hypoprolactinemia and with the consequent inability to breastfeed. Lactation has been shown to be positively affected by human recombinant prolactin (R-hPRL), which is able to increase serum PRL levels and milk volume in PRL-deficient women (221). Nevertheless, no further studies were published after 2011 about this topic, and R-hPRL is not commercially available nor routinely used in clinical practice.

It has been suggested to use a dopamine antagonist (such as domperidone or metoclopramide) in order to increase PRL levels and favour lactation. These drugs, however, are considered off-label and no recommendation regarding their routine use is available yet (222).

In general, given the low impact of this condition on the general health status, there are no further indications about the follow up.

## 8 Conclusions

As emerged from this review, the knowledge regarding isolated hormonal deficits of anterior pituitary gland is still lacking a full and wide characterization, from an epidemiological, etiological and therapeutical point of view. Although some deficits present a characteristic clinical picture that allows the clinicians to raise a prompt diagnostic suspicion (e.g., ICAI and IHH), other deficits present non-specific signs and symptoms leading to potential underdiagnosed conditions. This can result in a lower detection rate and underestimated incidence and prevalence. Furthermore, little is known about the possible causes of the alterations (e.g., genetic or environmental) that may be responsible for a late onset disease. Eventually, considering that some deficits can be life threatening or at least can lead to a worse quality of life, more awareness is needed to make a diagnosis and to start a proper treatment as early as possible.

## Author contributions

NP and SG conceived and designed the review. NP, LM, EV and DC conducted the literature search and wrote the first draft of the manuscript. MB, FB, CB, VG helped in writing the last version of the manuscript and supervised the whole work. All authors contributed to the article and approved the submitted version.
